# Strigolactones Decrease Leaf Angle in Response to Nutrient Deficiencies in Rice

**DOI:** 10.3389/fpls.2020.00135

**Published:** 2020-02-25

**Authors:** Masato Shindo, Shu Yamamoto, Koichiro Shimomura, Mikihisa Umehara

**Affiliations:** ^1^Graduate School of Life Sciences, Toyo University, Ora-gun, Japan; ^2^Department of Applied Biosciences, Toyo University, Ora-gun, Japan

**Keywords:** lamina joint, nitrogen deficiency, *Oryza sativa*, phosphate deficiency, strigolactone, sulfur deficiency

## Abstract

Strigolactones (SLs) are a class of plant hormones that are synthesized from *β*-carotene through sequential reactions catalyzed by DWARF (D) 27, D17, D10, and OsMORE AXILLARY GROWTH (MAX) 1 in rice (*Oryza sativa* L.). In rice, endogenous SL levels increase in response to deficiency of nitrogen, phosphate, or sulfate (−N, −P, or −S). Rice SL mutants show increased lamina joint (LJ) angle as well as dwarfism, delayed leaf senescence, and enhanced shoot branching. The LJ angle is an important trait that determines plant architecture. To evaluate the effect of endogenous SLs on LJ angle in rice, we measured LJ angle and analyzed the expression of SL-biosynthesis genes under macronutrient deficiencies. In the “Shiokari” background, LJ angle was significantly larger in SL mutants than in the wild-type (WT). In WT and SL-biosynthesis mutants, direct treatment with the SL synthetic analog GR24 decreased the LJ angle. In WT, deficiency of N, P, or S, but not of K, Ca, Mg, or Fe decreased LJ angle. In SL mutants, deficiency of N, P, or S had no such effect. We analyzed the time course of SL-related gene expression in the LJ of WT deficient in N, P, or S, and found that expression of SL-biosynthesis genes increased 2 or 3 days after the onset of deficiency. Expression levels of both the SL-biosynthesis and signaling genes was particularly strongly increased under −P. Rice cultivars “Nipponbare”, “Norin 8”, and “Kasalath” had larger LJ angle than “Shiokari”, interestingly with no significant differences between WT and SL mutants. In “Nipponbare”, endogenous SL levels increased and the LJ angle was decreased under −N and −P. These results indicate that SL levels increased in response to nutrient deficiencies, and that elevated endogenous SLs might negatively regulate leaf angle in rice.

## Introduction

Leaf angle is generally defined as the inclination between the leaf blade midrib and the stem, and is one of the most important plant architecture parameters that influence light interception, photosynthetic efficiency, and planting density (Mantilla-Perez and Salas Fernandez, 2017). Leaf erectness enhance light capture, improve photosynthetic assimilation, and help high density planting (Sinclair and Sheehy, 1999; Sakamoto et al., 2006). Thus, leaf angle is an important agricultural trait that contributes to grain yield in cereal crops. In rice (*Oryza sativa* L.), leaf angle is determined by the shape of the lamina joint (LJ), which connects the leaf blade and sheath (Hoshikawa, 1989).The lack of cell longitudinal elongation results in a small LJ angle, and cell elongation on the adaxial side of the LJ induces leaf blade bending away from vertical axis to a more horizontal position (Cao and Chen, 1995; Zhang et al., 2009; Zhao et al., 2010).

Rice LJ angle is regulated by plant hormones. Brassinosteroids (BR) stimulate elongation of adaxial parenchyma cells at the LJ and increase LJ angle (Wada et al., 1981; Cao and Chen, 1995; Zhang et al., 2009). BR also inhibit proliferation of abaxial sclerenchyma cells by controlling U-type cyclin CYC U4;1 (Sun et al., 2015). Gibberellin stimulates cell elongation, and interacts with BR signaling by several regulators (Shimada et al., 2006; Wang et al., 2009). Gibberellin reduced the leaf angle by inhibiting BR response, demonstrating that gibberellin is a negative regulator of lamina inclination (Tong et al., 2014). Auxin also increases LJ angle *via* a BRI1-dependent and -independent pathway (Nakamura et al., 2009; Zhao et al., 2013; Zhang et al., 2015). In contrast, methyl jasmonate represses BR biosynthesis and signaling, and reduces LJ angle (Gan et al., 2015). Recently, it was reported that leaf inclination is higher in strigolactone (SL) mutants than in wild type (WT), and that exogenously applied GR24, a synthetic SL analog, decreases the large inclination in SL-biosynthesis mutants (Li et al., 2014). However, how LJ angle is regulated by SL signal remains unknown.

SL was originally isolated from plant root exudates as a seed germination stimulant of witchweed (Cook et al., 1966; Cook et al., 1972). Later, SL was identified as a stimulator of hyphal branching in arbuscular mycorrhizal fungi, which supply soil nutrients to host plants (Akiyama et al., 2005). The previous studies proposed that SL functions as a communication signal for parasitism and symbiosis in the rhizosphere (Bouwmeester et al., 2007). More later, SLs were found as a class of phytohormones that inhibit shoot branching (Gomez-Roldan et al., 2008; Umehara et al., 2008). In this time, SLs are also known to control root architecture and promote leaf senescence, endosperm development, and secondary growth (Agusti et al., 2011; Kapulnik et al., 2011; Ruyter-Spira et al., 2011; Yamada et al., 2014; Ueda and Kusaba, 2015; Yamada et al., 2018).

In rice, SL-biosynthesis mutants *d27*, *d17*/*high tillering dwarf* (*htd*)*1*, and *d10* are well characterized. *D27*, *D17*/*HTD1*, and *D10* encode β-carotene isomerase, carotenoid cleavage dioxygenase (CCD) 7, and CCD8, respectively (Zou et al., 2006; Arite et al., 2007; Lin et al., 2009). In rice SL biosynthesis, all-*trans*-*β*-carotene is converted to carlactone by sequential reactions *via* D27, D17, and D10 (Alder et al., 2012; Seto et al., 2014); carlactone is converted to SL by MAX1, which is a cytochrome P450 CYP711 family protein (Zhang et al., 2014). There are five MAX1 homologs in rice (Nelson et al., 2004); among them, Os900 converts carlactone to 4-deoxyprobanchol *via* carlactonoic acid, and Os1400 converts carlactone to 4-deoxyprobanchol (4DO), and 4DO to orobanchol (Yoneyama et al., 2018a). The SL signaling mutant *d14* has a mutation in an α/β-hydrolase protein (Arite et al., 2009). D14 protein has a dual function as a receptor and deactivator of bioactive SLs (Seto et al., 2019). Another SL signaling mutant, *d3*, has a mutation in leucine-rich-repeat F-box protein (Ishikawa et al., 2005), which acts as a recognition subunit in the SKP-CUL1-F-box (SCF) protein complex, binds target proteins and directs them for proteasomal degradation. The SL-insensitive mutant *d53* has a mutation in a repressor of SL signaling (Jiang et al., 2013; Zhou et al., 2013). D53 is the target protein of the SCF^D3^ ubiquitination complex in SL signaling.

SLs are produced mainly in roots in response to nitrogen and/or phosphate deficiencies in several plant species (Yoneyama et al., 2007a; Yoneyama et al., 2007b; Lopez-Raez et al., 2008; Yoneyama et al., 2012). In a rice cultivar “Shiokari”, SLs are produced in response to nitrogen, phosphate, and sulfur deficiencies (Umehara et al., 2010; Sun et al., 2014; Shindo et al., 2018). In this study, we investigated the effect of nutrient deficiencies on endogenous SL production and on LJ angle in rice. Furthermore, we compared the LJ angles of WT and *d* mutants grown under nutrient-deficient or sufficient conditions, analyzed SL-related gene expression, and measured canonical SL levels in roots.

## Materials and Methods

### Plant Growth Conditions and Measurement of LJ Angle

In this study, we used wild-type (WT) plants and *dwarf* mutants *d27-1*, *d17-1*, *d10-1*, *d14-1*, and *d3-1* in the “Shiokari” background, *d10-2*, *d17-2* in the “Nipponbare” background, and *d53* in the “Norin 8” background (Ishikawa et al., 2005; Arite et al., 2007; Umehara et al., 2008; Arite et al., 2009; Lin et al., 2009; Zhou et al., 2013; Kobae et al., 2018). The seeds were kindly provided by Prof. Junko Kyozuka (Tohoku University) and by Dr. Hiroaki Iwai and were propagated in a glass room at the Research Center for Life and Environmental Sciences, Toyo University, Japan. Rice seedlings were grown hydroponically as described previously (Umehara et al., 2008). Surface-sterilized seeds were incubated in sterile water at 25°C in the dark for 1 day, and germinated seeds were transferred to hydroponic culture medium (Kamachi et al., 1991) solidified with 0.6% agar and cultured under 16 h fluorescent white light (130–180 µmol m^−2^ s^−1^) at 25°C and 8 h dark at 23°C for 6 days (pre-culture). Seedlings of similar size were then transferred to hydroponic medium containing 1 mM 2-(*N*-morpholino) ethanesulfonic acid (MES) buffer (pH 5.7) for a further 3, 6, or 24 days. Seedlings were placed in soil and cultivated in a glass room for 34 days. LJ angle (between leaf blade and sheath) of the 2nd, 3rd, and 4th leaves was measured on leaf images in ImageJ software v. 1.50 (Schneider et al., 2012).

### Chemicals

*rac*-GR24, a synthetic SL analog, was purchased from Chiralix (Nijmegen, The Netherlands) and dissolved in acetone. Mock solution or 20 μM *rac*-GR24 containing 0.1% Tween-20 were spotted (2 μl) onto the LJ of WT and *d* mutants on days 0 and 3 of cultivation in hydroponic medium. The following standards were used for SL quantification: 4DO, *d*_1_-labeled 4DO, *d*_3_-labeled 4DO, orobanchol, *d*_1_-labeled orobanchol, and *d*_3_-labeled orobanchol; all were kindly provided by Prof. Kohki Akiyama (Osaka Prefecture University).

### RNA Extraction and qRT-PCR

Total RNA was extracted from approximately 25 mg LJ segments using an RNeasy Plant mini kit (Qiagen, Hilden, Germany) following the instructions in the user manual. We used 0.1 μg of the total RNA for cDNA synthesis with a ReverTra Ace qPCR RT kit for quantitative real-time PCR (qRT-PCR) (Toyobo, Osaka, Japan). qRT-PCR was performed in a StepOnePlus thermocycler (Thermo Fisher Scientific, Waltham, MA, USA) with a Thunderbird Probe qPCR mix (Toyobo). Expression of *ubiquitin* was used as an internal standard. Expression levels of SL-related genes were quantified using the specific primers and probes used in a previous study (Shindo et al., 2018).

### SL Purification and Quantification

We measured the levels of 4DO and orobanchol in roots as described previously (Hasegawa et al., 2018; Shindo et al., 2018) with some modifications. Germinated seeds were pre-cultured in agar culture media for 7 days and the seedlings were grown in hydroponic culture media for a further 7 days. Roots (ca. 1 g) of 14-day-old seedlings were homogenized in 10 ml acetone containing 100 pg each of *d*_1_-labeled 4DO and orobanchol as internal standards; the homogenates were filtered with Bond Elute reservoirs (Agilent, Santa Clara, CA, USA) and evaporated to dryness under nitrogen gas. The extracts were dissolved in 4 ml water adjusted to pH 2–3 with 1 N HCl and extracted twice with 4 ml ethyl acetate. The ethyl acetate phase was evaporated to dryness under nitrogen gas. The extracts were dissolved in 10% acetone, loaded onto Oasis HLB 3-ml cartridges (Waters), washed with 10% acetone, and eluted with 60% acetone. The eluates were dissolved in ethyl acetate: *n*-hexane (15:85) and loaded onto Sep-Pak Silica 1-ml cartridges (Waters). After the cartridges were washed with ethyl acetate: *n*-hexane (15:85), 4DO was eluted with ethyl acetate: *n*-hexane (35:65) and orobanchol with ethyl acetate: *n*-hexane (50:50). LJ sample (ca. 0.15 g) were homogenized in 10 ml acetone containing 100 pg each of *d*_3_-labeled 4DO and orobanchol; the homogenates were filtered with Bond Elute reservoirs (Agilent, City CA, USA) and evaporated to dryness under nitrogen gas. The extracts were dissolved in 4 ml 50% acetonitrile adjusted to pH 2–3 with 1 N HCl, and 4 ml hexane, mixed gently, centrifuged at 3,400 rpm for 5 min, and the hexane-phase was removed. After the aqueous-phase was extracted with 4 ml ethyl acetate twice, 4DO, and orobanchol in the ethyl acetate-phase were purified according to the method in roots.

Purified SL-containing fractions were dissolved in 50% acetonitrile and subjected to liquid chromatography–tandem mass spectrometry (LC-MS/MS) analysis using a system consisting of a quadrupole tandem mass spectrometer (3200 QTRAP; Sciex, Framingham, MA, USA) and a high-performance liquid chromatograph (Prominence, Shimadzu, Kyoto, Japan) equipped with a reverse-phase column (Acquity UPLC BEH-C18, 2.1 × 50 mm, 1.7 µm, Waters). Previous papers describe LC and MS parameters for 4DO (Shindo et al., 2018) and orobanchol (Hasegawa et al., 2018) analysis. Data were analyzed in Analyst 1.5.1 and Multi Quant 2.0.2 (Sciex) software.

### Statistical Analysis

Statistical analysis was performed in SPSS 24 software (IBM SPSS Inc., Armonk, NY USA). Student's *t*-test (*P* < 0.05) was used for pairwise comparisons and Tukey's honestly significant difference (HSD) (*P* < 0.05) for multiple comparisons.

## Results

### LJ angle in Rice SL Mutants in “Shiokari” Background

**Figure 1:**
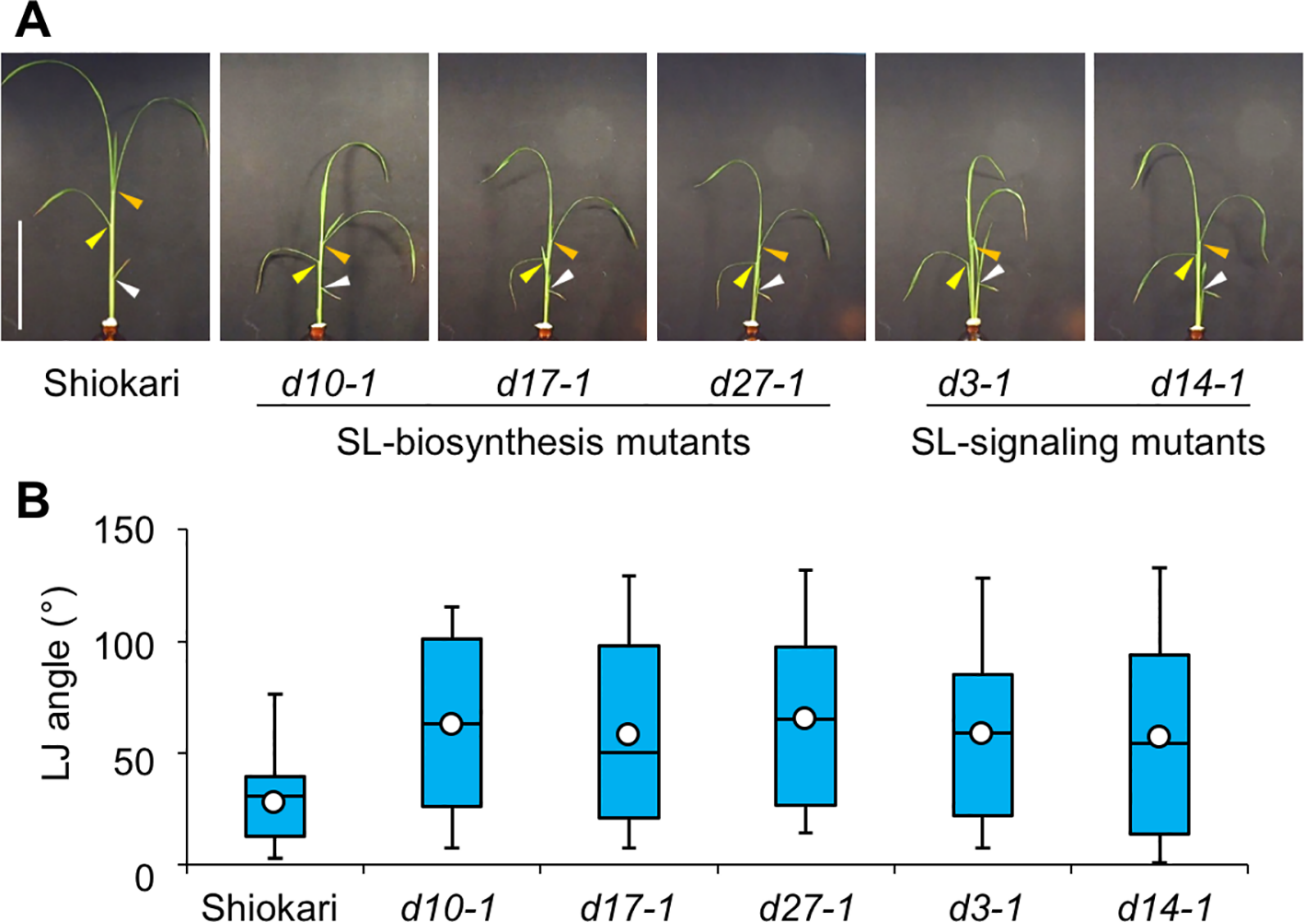
Lamina joint (LJ) angle in “Shiokari”-background wild-type and strigolactone (SL) mutant rice seedlings. **(A)** Images of 30-d-old seedlings. Arrowheads: white, 2nd LJ; yellow, 3rd LJ; orange, 4th LJ. Bar: 10 cm. **(B)** LJ angle measured with ImageJ software. White circles indicate average (*n* = 60).

**Figure 2:**
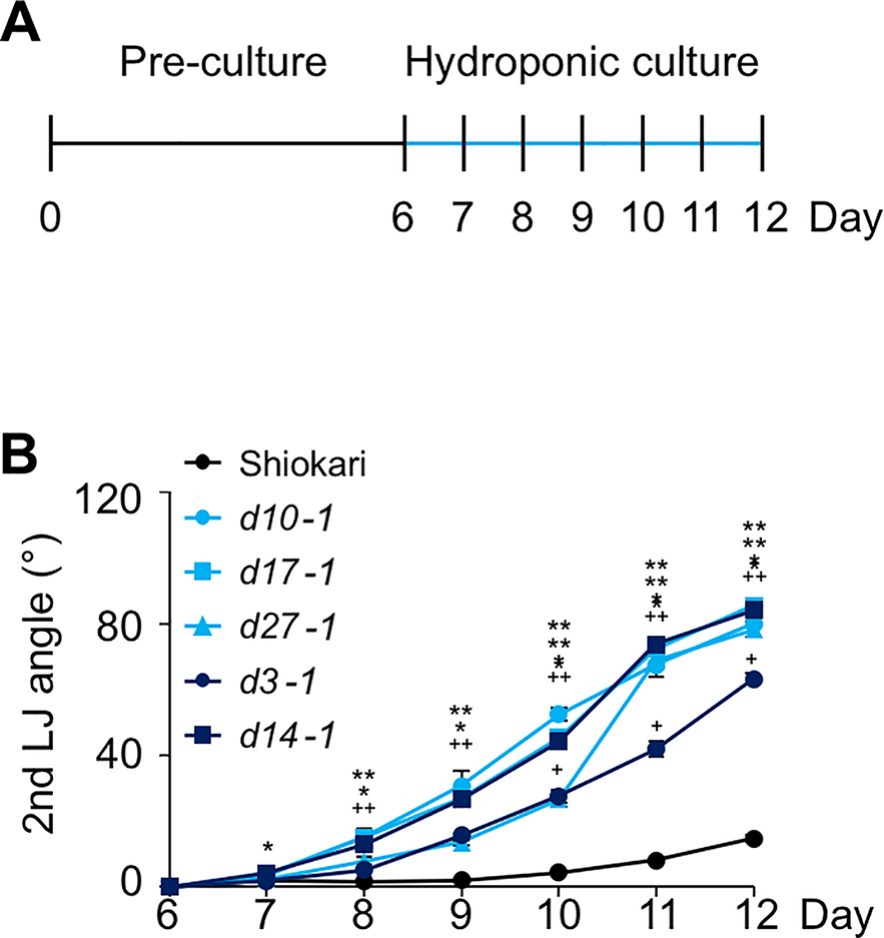
Time-course analysis of LJ angle in the 2nd leaf of rice seedlings. **(A)** Timeline of the experiment. After pre-culture, seedlings were transferred to nutrient-sufficient hydroponic medium. **(B)** LJ angle of wild-type “Shiokari” and SL mutants. Asterisks and plus signs indicate significant differences in ANOVA (all *P* < 0.05) vs. ‘Shiokari’. Data are means ± S.E. (*n* = 5).

Many SL-biosynthesis and signaling mutants are available in the background of the rice cultivar ‘Shiokari'. The 2nd, 3rd, and 4th leaves of the seedlings that had been grown hydroponically for a month are shown in **Figure 1A**. The LJ angle of SL mutants was almost twice as large as that of WT ‘Shiokari' (**Figure 1B**). In seedlings grown in soil for 40 days, it was slightly smaller, but that of SL mutants was also almost twice as large as that of WT (**Supplementary Figure S1**), indicating that the LJ angle of SL mutants was increased regardless of the growth conditions. To simplify the comparison between WT and SL mutants, we focused on the 2nd LJ angle. After pre-culture for 6 days (**Figure 2A**), the 2nd LJ angle was 0°, with no significant difference between WT and SL mutants (**Figure 2B**). However, it became larger in SL mutants than in WT on day 7, continued to increase from day 8 to day 11, and reached maximum in both WT and SL mutants on day 12 (**Figure 2B**). In a previous study (Li et al., 2014), the LJ angle was greater in *d3-1* than in other *d* mutants, but in our experimental conditions, it was smaller in *d3-1* (**Figure 2B**). We confirmed that exogenously applied 20 µM GR24 (SL synthetic analog) strongly decreased LJ angle in WT and the SL-biosynthesis mutants *d10-1*, *d17-1*, and *d27-1*, but had no effect on the SL-signaling mutants *d3-1* and *d14-1* (**Supplementary Figure S2**).

### Effects of Macronutrient Deficiencies on LJ Angle, SL-Related Gene Expression, and SL Levels in the “Shiokari” Background

**Figure 3:**
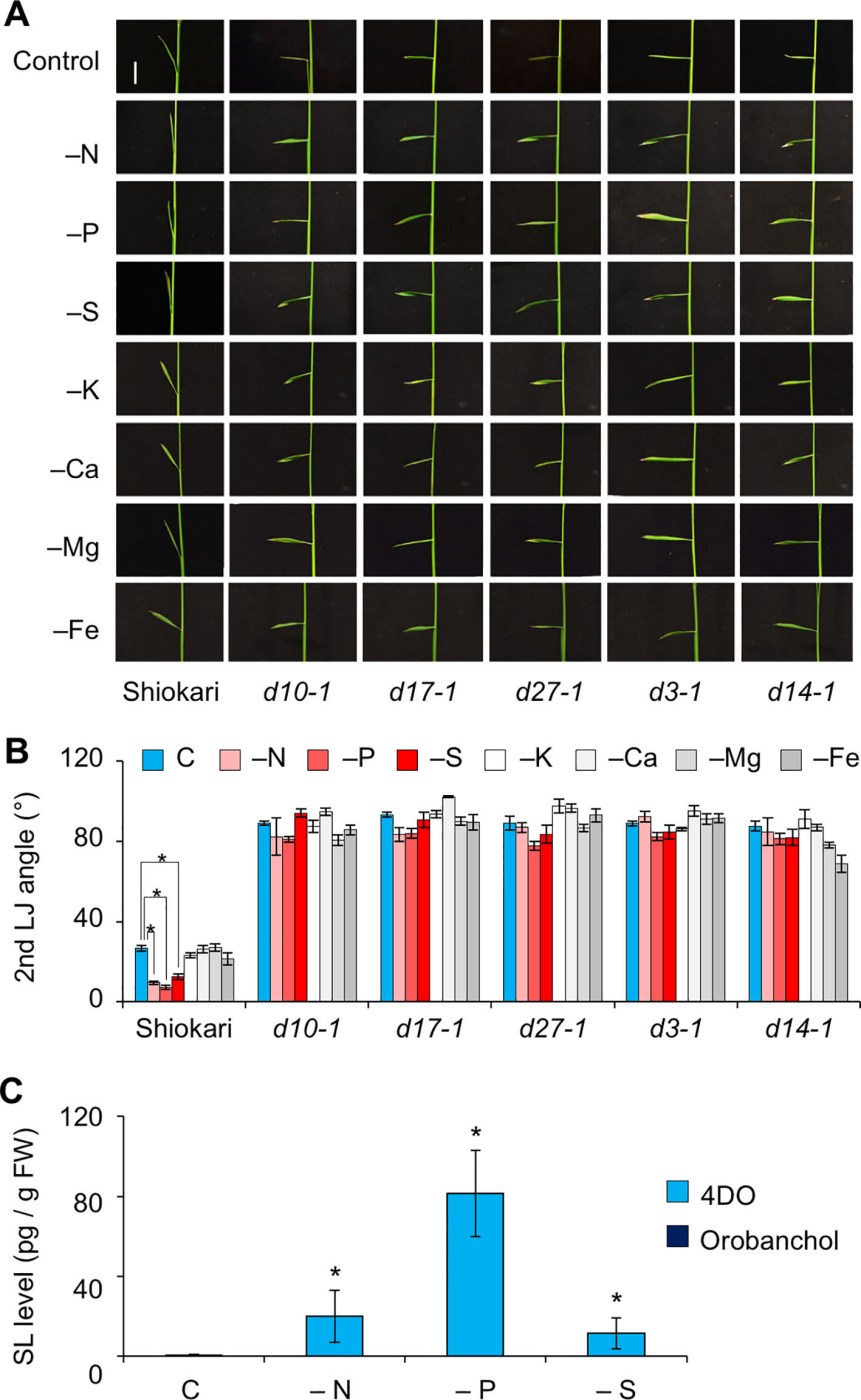
Effect of macronutrient deficiency on LJ angle and SL levels in “Shiokari”-background seedlings. **(A)** Images of the 2nd leaves of 12-d-old seedlings. Bar, 1 cm. **(B)** Second-LJ angle under macronutrient deficiencies. C, control. *P < 0.05 (Student's *t*-test). Data are means ± S.E. (*n* = 5; eight seedlings per experiment). **(C)** SL levels in roots under nitrogen, phosphate, or sulfate deficiency in roots of 14-d-old seedlings. C, control. *P < 0.05 (Student's *t*-test vs. control). Data are means ± S.E. (*n* = 4).

In “Shiokari”, SL production in roots is stimulated in response to deficiency of nitrogen, phosphate, or sulfate (Shindo et al., 2018). To evaluate the effects of macronutrient deficiencies on LJ angle, we grew WT and SL mutant seedlings under deficiencies of nitrogen, phosphate, sulfate, potassium, calcium, or magnesium (−N, −P, −S, −K, −Ca, −Mg, and −Fe). The LJ angle of WT decreased by almost half under −N, −P, and −S in comparison with the control, whereas that of SL mutants was not affected by a deficiency of any of these macronutrients (**Figures 3A, B**). Using LC-MS/MS, we analyzed the levels of canonical SLs, 4DO and orobanchol, in roots. The 4DO levels in roots increased under −N, −P, and −S, but the levels of orobanchol were below the detection limit in “Shiokari” (**Figure 3C**).

**Figure 4:**
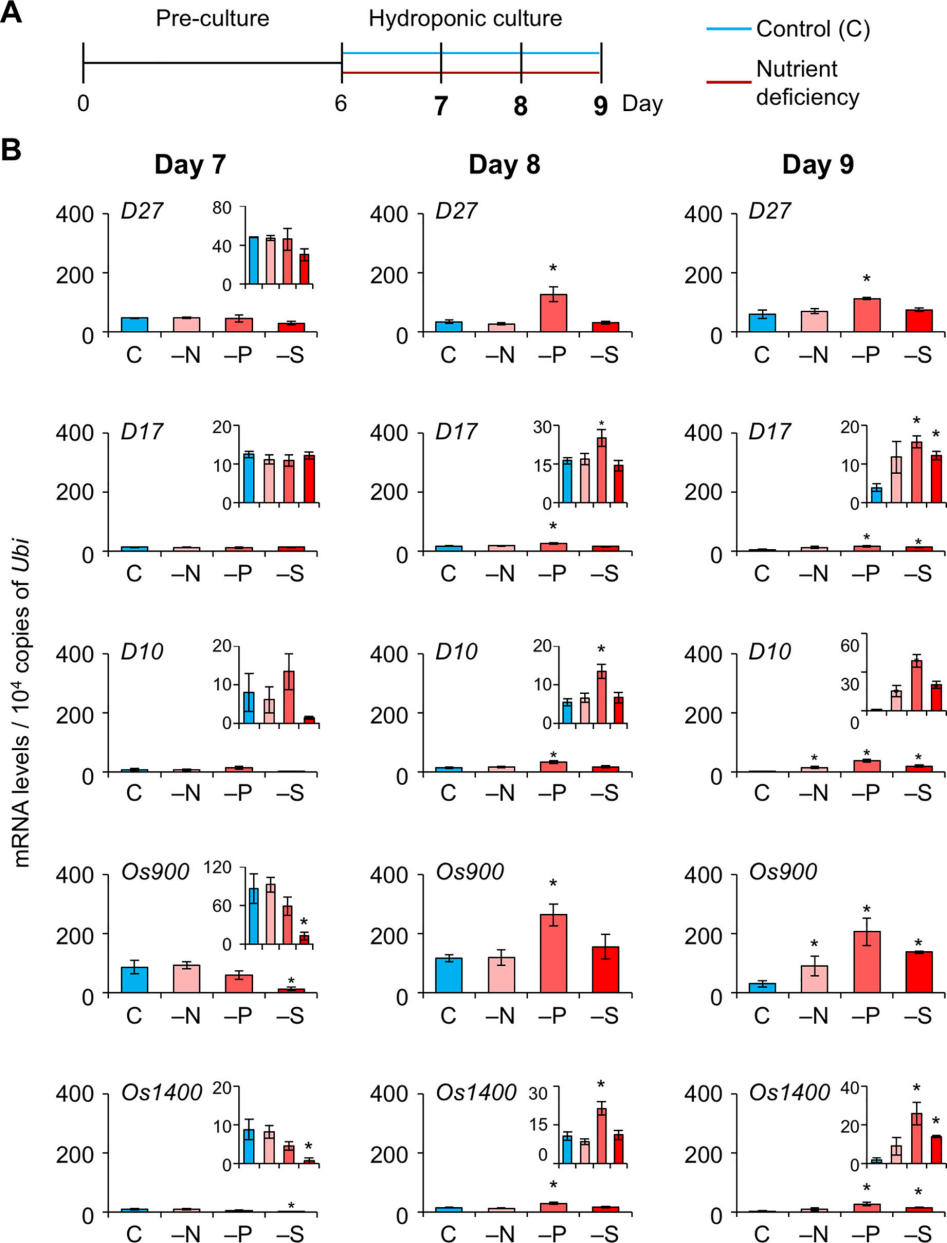
Expression of SL-biosynthesis genes in LJ under nitrogen, phosphate, or sulfate deficient condition. **(A)** Timeline of the experiment. After pre-culture, seedlings were transferred to control or nutrient-deficient hydroponic medium. Gene expression was analyzed on days 7, 8, and 9. **(B)** Transcript levels in the 2nd LJ. C, control. *P < 0.05 (Student's *t*-test vs. control). Data are means ± S.E. (*n* = 3, six seedlings per experiment).

**Figure 5:**
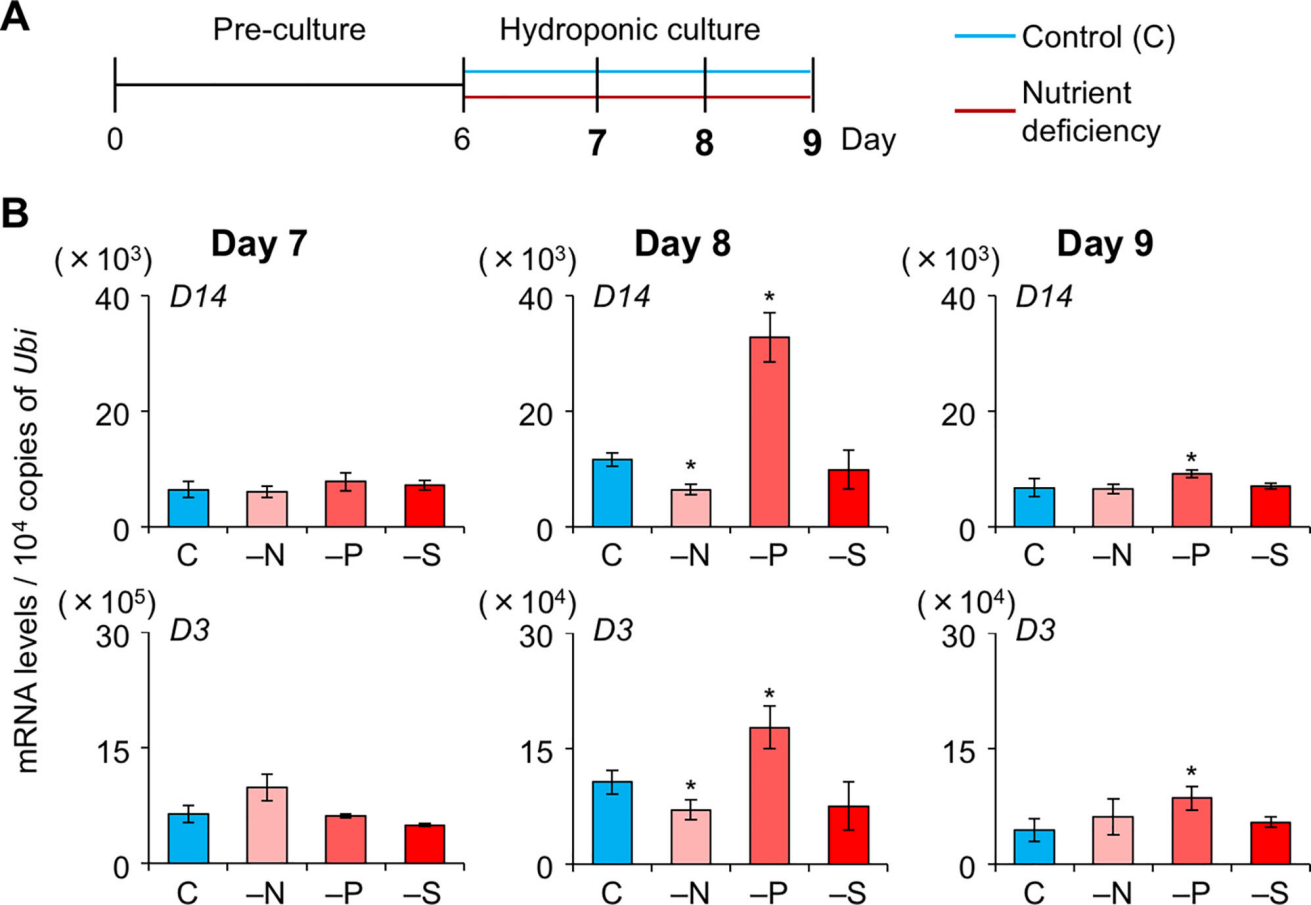
Expression of SL-signaling genes in LJ under nitrogen, phosphate, or sulfate deficient condition. **(A)** Timeline of the experiment. After pre-culture, seedlings were transferred to control or nutrient-deficient hydroponic medium. Gene expression was analyzed on days 7, 8, and 9. **(B)** Transcript levels in the 2nd LJ. C, control. *P < 0.05 (Student's *t*-test vs. control). Data are means ± S.E. (*n* = 3, six seedlings per experiment).

To explore whether SL levels increase in LJ under −N, −P, and −S, we analyzed the expression levels of SL-biosynthesis and signaling genes on days 7, 8, and 9, i.e. before a large increase in LJ angle (**Figure 4A**). On day 7, the expression of *OsMAX1* decreased under −S (**Figure 4B**). On day 8, the expression of all SL-biosynthesis genes under −P increased, whereas that of *OsMAX1* under −S returned to the control levels (**Figure 4B**). On day 9, the expression of some SL-biosynthesis genes increased under −N, −S, and −P (**Figure 4B**). All SL-biosynthetic genes we analyzed were up-regulated in 8-day-old seedlings grown under −P. Thus, we tried to analyze the levels of canonical SLs, 4DO and orobanchol, in LJ of the seedlings. However, we were unable to detect 4DO or orobanchol in LJ (**Supplementary Figure S3**). Among SL-signaling genes, the expression of *D3* and *D14* also increased under −P on days 8 and 9 (**Figure 5**). Expression of these genes slightly but significantly decreased under −N on day 8.

### LJ Angle and Response of Nutrient Deficiencies in Different Rice Cultivars

**Figure 6:**
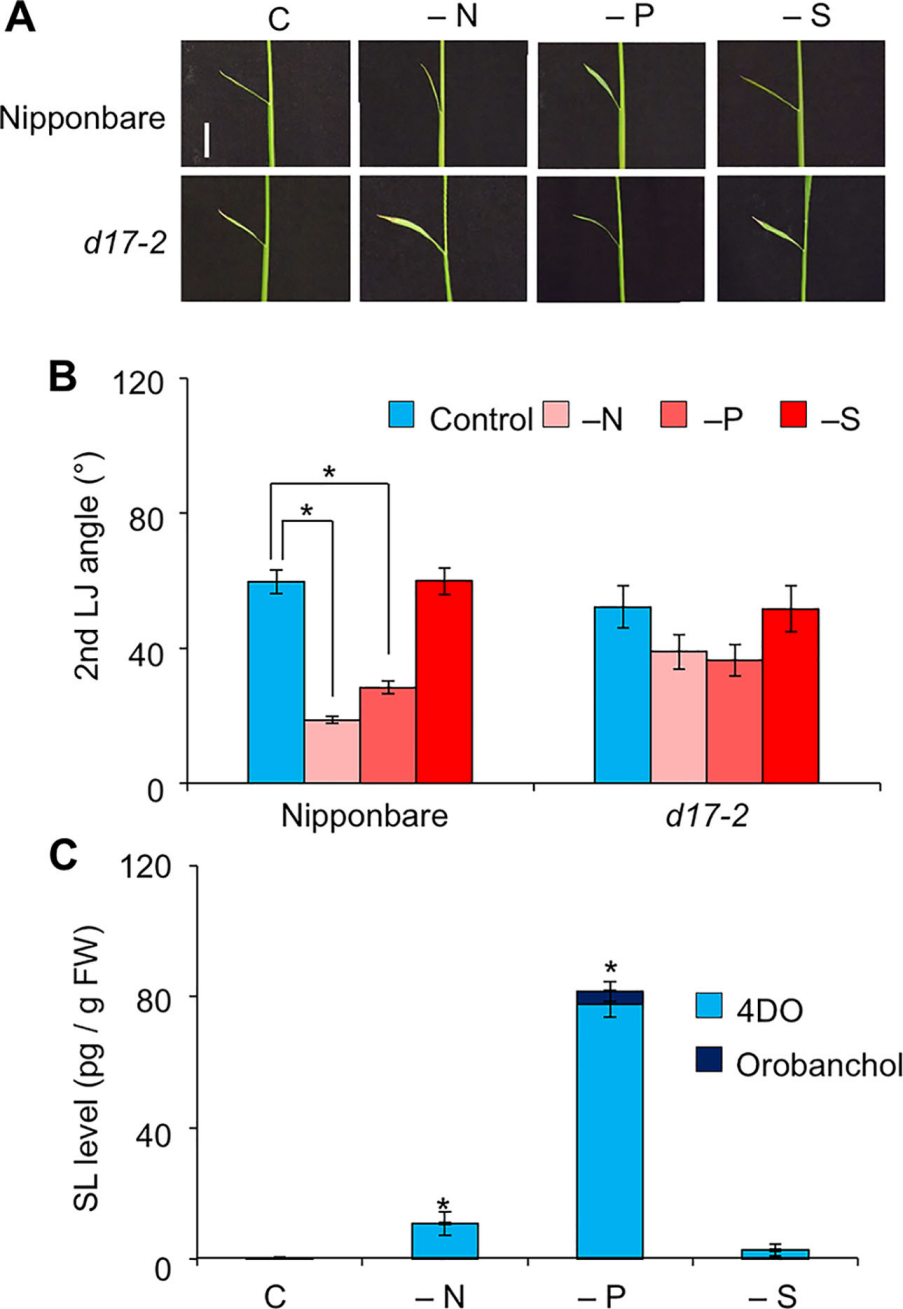
Effect of macronutrient deficiency on LJ angle and SL levels in “Nipponbare”-background rice seedlings. **(A)** Images of the 2nd leaves. Bar, 1 cm. **(B)** Second-LJ angle under macronutrient deficiencies. C, control. *P < 0.05 (Student's *t*-test). Data are means ± S.E. (*n* = 5). **(C)** SL levels in roots under nitrogen, phosphate, or sulfate deficiency in roots of 14-d-old seedlings. C, control. *P < 0.05 (Student's *t*-test vs. control). Data are means ± S.E. (*n* = 4).

The LJ angle was smaller in “Shiokari” than in “Nipponbare”, “Norin 8”, and “Kasalath” (**Supplementary Figures S4A, B**). It did not differ significantly between WT “Nipponbare” and the *d10-2* and *d17-2* mutants in the “Nipponbare” background (**Supplementary Figure S4C**), or between WT “Norin 8” and *d53* in the “Norin 8” background (**Supplementary Figure S4D**). In WT “Nipponbare”, it decreased by half under −N and −P, but did not change under −S (**Figures 6A, B**). That of *d17-2* did not change under −N, −P, or −S (**Figures 6A, B**). In “Nipponbare” roots, 4DO levels increased under −N and especially −P and orobanchol was only detected under −P (**Figure 6C**). Unlike in “Shiokari” (**Figure 3C**), we detected no significant SL increase under −S in “Nipponbare”. Exogenously applied 20 µM GR24 decreased LJ angle in WT and the SL-biosynthesis mutants *d17-2* (**Supplementary Figure S5**).

## Discussion

**Figure 7:**
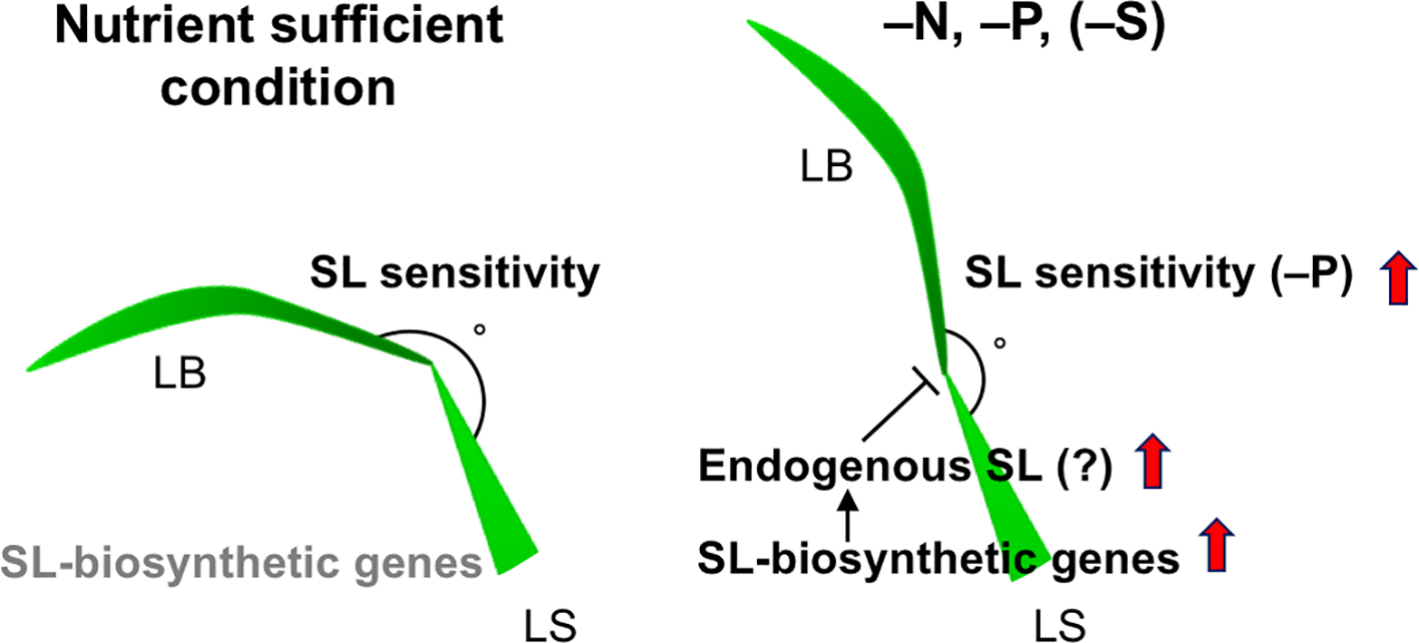
A proposed model on LJ angle regulation through SL signals. Expression levels of SL-biosynthetic genes are low in nutrient sufficient condition, whereas they are up-regulated in –N, –P (–S in Shiokari), probably stimulating endogenous SL production in LJ. SL sensitivity of LJ is basically high, but the sensitivity is increased in –P. LB, leaf blade, LS, lead sheath.

We investigated the effects of SLs on LJ angle under macronutrient deficiencies in rice. In response to −N, −P, and −S, SL-biosynthesis genes were upregulated and endogenous SL levels increased, decreasing LJ angle in “Shiokari” (**Figures 3**, **4**, and **7**). Phosphate deficiency had the strongest effect on expression of SL-biosynthesis genes in LJ and SL production in roots (**Figures 3** **and** **4**). The results are consistent with our previous report that SL levels increase in response to −N, −P, and −S in “Shiokari” (Shindo et al., 2018). Expression of SL-biosynthesis genes in −P were higher than that in −N and −S, but the LJ angle of rice seedlings under -N and -S is narrow at the same level as that of -P plants (**Figures 3** and **4**). Expression of SL-signaling genes, *D3* and *D14*, were higher than that of SL-biosynthesis genes (**Figures 4** and **5**). These results suggest that LJ angle might be sensitive to endogenous SL (**Figure 7**). In addition, *D3* and *D14*, were up-regulated under −P, indicating that sensitivity to endogenous SLs also increased under −P (**Figure 5**). Phosphate deficiency induces *Syg1/Pho81/XPR1* (*SPX1*) and *SPX2* expression, which inhibit *REGULATOR OF LEAF INCLINATION* (*RLI1*), a positive regulator of leaf inclination, reducing leaf angle *via* suppression of downstream BR signaling (Ruan et al., 2018). Under low N, leaf angle is reduced in rice and eucalyptus to avoid damage by photoinhibition (Close and Beadle, 2006; Kumagai et al., 2014). Under nutrient deficiencies, rice grows slowly and has fewer tillers, lower chlorophyll content, and lower yield (Dobermann and Fairhurst, 2001). The decrease in leaf chlorophyll content leads to production of reactive oxygen species, which attack cell components (Jia et al., 2003). High-intensity light causes photoinhibition of photosynthesis (Powles, 1984), although it also increases SL production in tomato roots (Koltai et al., 2011). Therefore, plants might decrease LJ angle to avoid photoinhibition *via* SL signals produced under −N, −P, or −S. Previously we suggested that plants use SL signaling to utilize limited nutrients efficiently and to adapt to poor nutrition because SLs are produced under −N and/or −P (Umehara, 2011). Here, we propose that SLs are important for photosynthetic efficiency and nutrient allocation.

We could not find rice canonical SLs, 4DO, or orobanchol, in the LJ even under −P when multiple SL genes were highly expressed (**Figure 4**, **Supplementary Figure S3**). In rice, SLs regulating the LJ angle might be non-canonical SLs such as carlactonoic acid and methyl carlactonoate (Yoneyama et al., 2018b). Canonical SLs might be transported from roots to the LJ and converted to active signaling molecules during the transportation, or induce other active signaling molecule.

Rice SL-related mutants of “Shiokari” background have increased LJ angle (Li et al., 2014). We found such phenotypes in SL mutants in the “Shiokari” background (**Figures 1** and **2**). The phenotype of the SL-related mutants depends on the cultivar: the LJ angles in “Nipponbare”, “Norin 8”, and “Kasalath” were twice that of “Shiokari”, with no significant differences between WT and SL mutants (**Supplementary Figure S4**). In response to −N and −P, SL production was stimulated and the LJ angle decreased in “Nipponbare”, but not in *d17-2* (**Figure 6**). This point was common between “Shiokari” and “Nipponbare”. Thus, endogenous SL levels would increase in response to nutrient deficiencies and decrease leaf angle in rice. In the future, which types of SLs directly regulate the leaf angle would be clarified.

Auxin, GA, and BR also regulate LJ angle. The LJ angle was larger in SL mutants in the “Shiokari” background than in WT “Shiokari” (**Figure 1**), indicating that BR might accumulate in the LJ of the SL mutants or SL might positively regulate BR signaling. Interaction between SL and these phytohormones is well characterized in shoot branching regulation. In the BR signaling pathway, BRI-EMS-suppressor 1 (BES1) acts as a downstream transcription factor to positively regulate BR-responsive gene expression (Yin et al., 2002). In Arabidopsis, BES1 binds to MAX2, an ortholog of rice D3, and the complex is degraded to inhibit shoot branching (Wang et al., 2013). Auxin stimulates SL biosynthesis, whereas SL regulates polar auxin transport, contributing to shoot branching regulation (Domagalska and Leyser, 2011). Gibberellin inhibits SL biosynthesis in rice (Ito et al., 2017). The position of SL in this phytohormone cross-talk of LJ angle regulation remains unknown. Further analysis would be required to determine the interactions between SL and other phytohormones.

## Data Availability Statement

All datasets generated for this study are included in the article/**Supplementary Material**.

## Author Contributions

MU and KS designed the research. MS and SY performed the experiments and analyzed the data. MS and MU wrote the paper.

## Funding

This work was in part supported by a grant from Toyo University (the Inoue Enryo Memorial Foundation for Promoting Sciences to MS), from Ichimura Foundation for New Technology (the Plant Research Grant, No. 28-19 to MU), and from the Japan Society for the Promotion of Science (KAKENHI, No. 17K07650 to MU).

## Conflict of Interest

The authors declare that the research was conducted in the absence of any commercial or financial relationships that could be construed as a potential conflict of interest.
