# Supplementary material for: Strigolactones Decrease Leaf Angle in Response to Nutrient Deficiencies in Rice
Source: Front Plant Sci. 2020 Feb 25;11:135. doi: 10.3389/fpls.2020.00135 (PMC7052320; doi:10.3389/fpls.2020.00135)
Supplement: Supplementary file 1 [file DataSheet_1.pdf]

Fig. S1

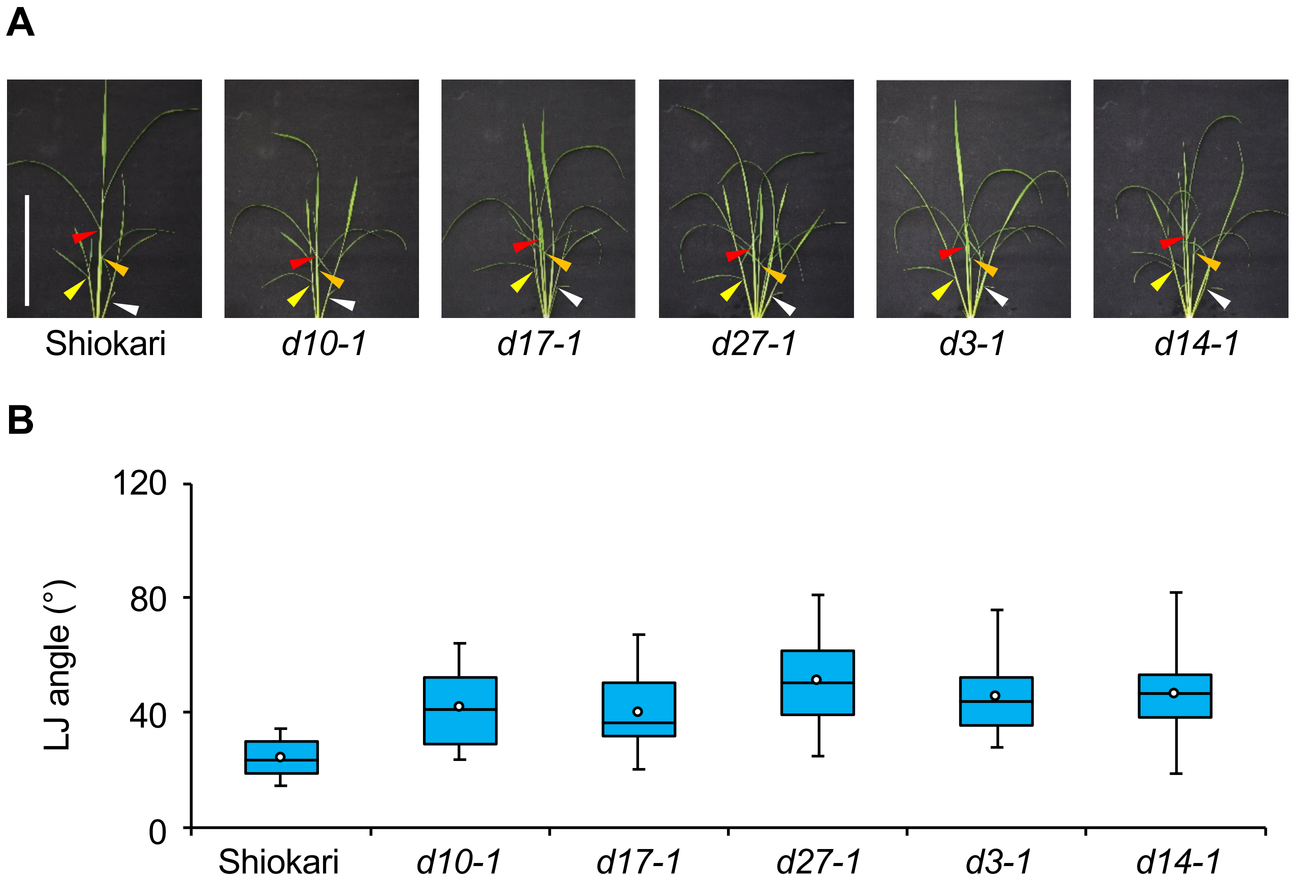

FIGURE S1 | LJ angle of ‘Shiokari’-background rice seedlings grown in soil. **(A)** Images of the 2nd, 3rd, and 4th leaves of 40-day-old plants. Arrowheads: white, 2nd LJ; yellow, 3rd LJ; orange, 4th LJ. Bar, 10 cm. **(B)** LJ angles measured with ImageJ. White circles indicate average (*n* = 24).

Fig. S2

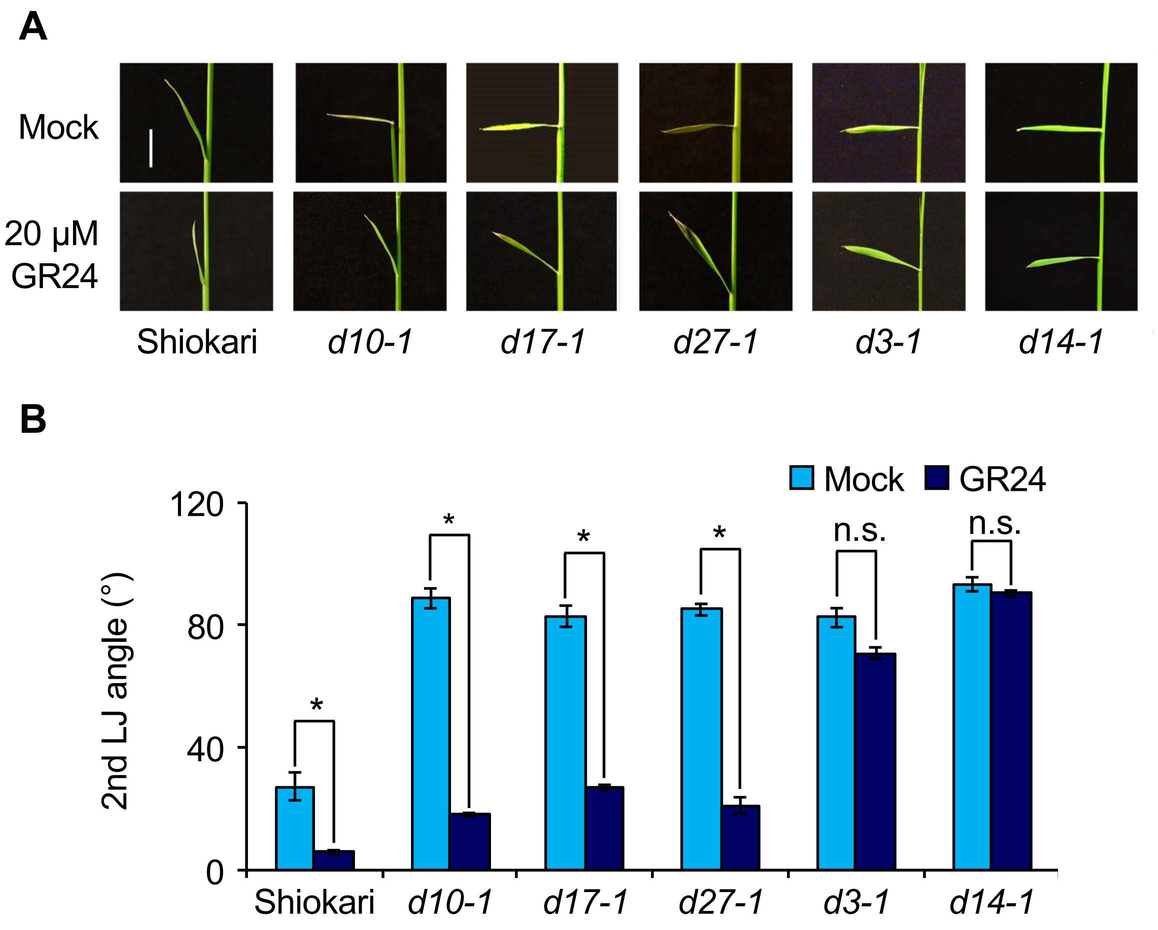

FIGURE S2 | Effect of exogenously applied GR24 on LJ angle in 'Shiokari'-background rice seedlings. **(A)** Images of the 2nd leaves of 12-day-old seedlings treated or not with 20 μM GR24. Bar, 1 cm. **(B)** Second-LJ angle of seedlings treated or not with GR24. *$P < 0.05$ (Student's *t*-test). n.s., not significant. Data are means ± S.E. ($n = 5$, eight seedlings per experiment).

Fig. S3

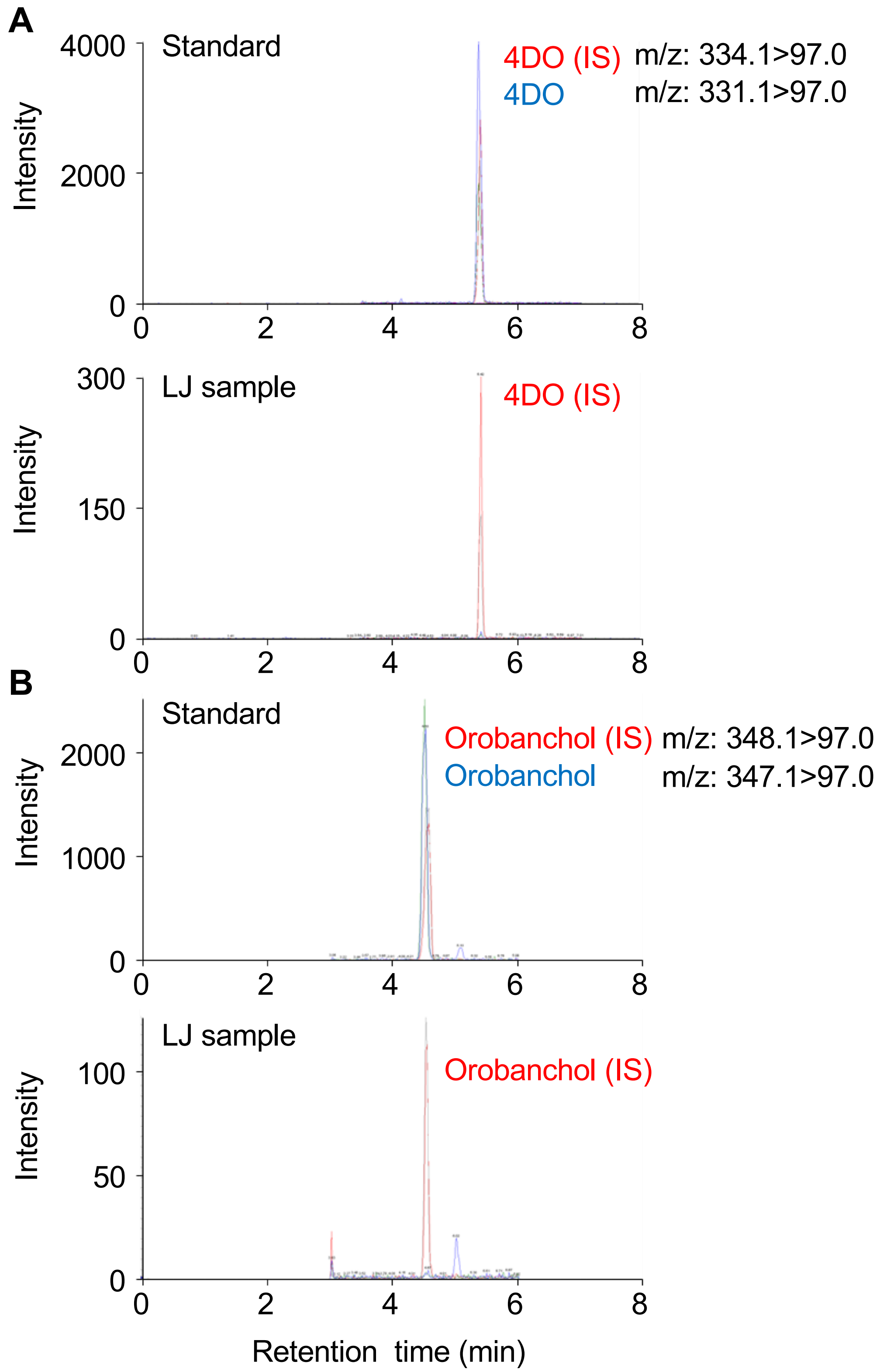

FIGURE S3 | Chromatograms of canonical SLs in LC-MS/MS analysis. Blue peaks, authentic standard; red peaks, internal standard (IS). **(A)** 4DO; **(B)** orobanchol. Endogenous 4DO and orobanchol were not detected in LJ samples of 8-day-old seedlings grown under −P .

Fig. S4

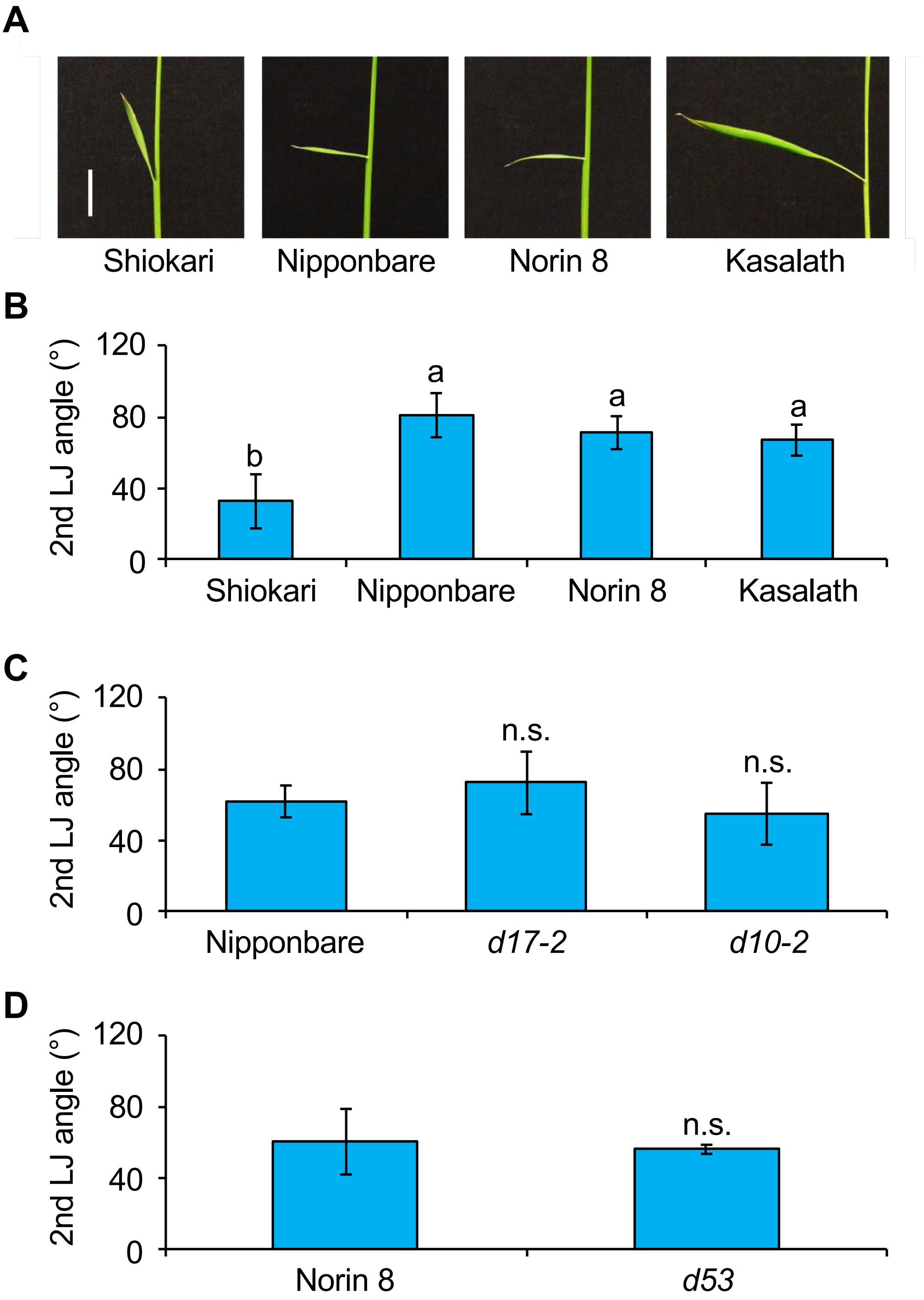

FIGURE S4 | Differences in LJ angle between rice cultivars. **(A)** Images of the 2nd leaves of 12-day-old seedlings. Bar, 1 cm. **(B)** Second-LJ angle measured with ImageJ. Different letters indicate significant differences in Tukey's HSD ($P < 0.05$). **(C, D)** Second-LJ angle in 12-day-old wild-type and SL-mutant seedlings with the 'Nipponbare' **(C)** and 'Norin 8' **(D)** background. n.s., not significant (Student's *t*-test vs. control). All data are means ± S.D. ($n = 8$).

Fig. S5

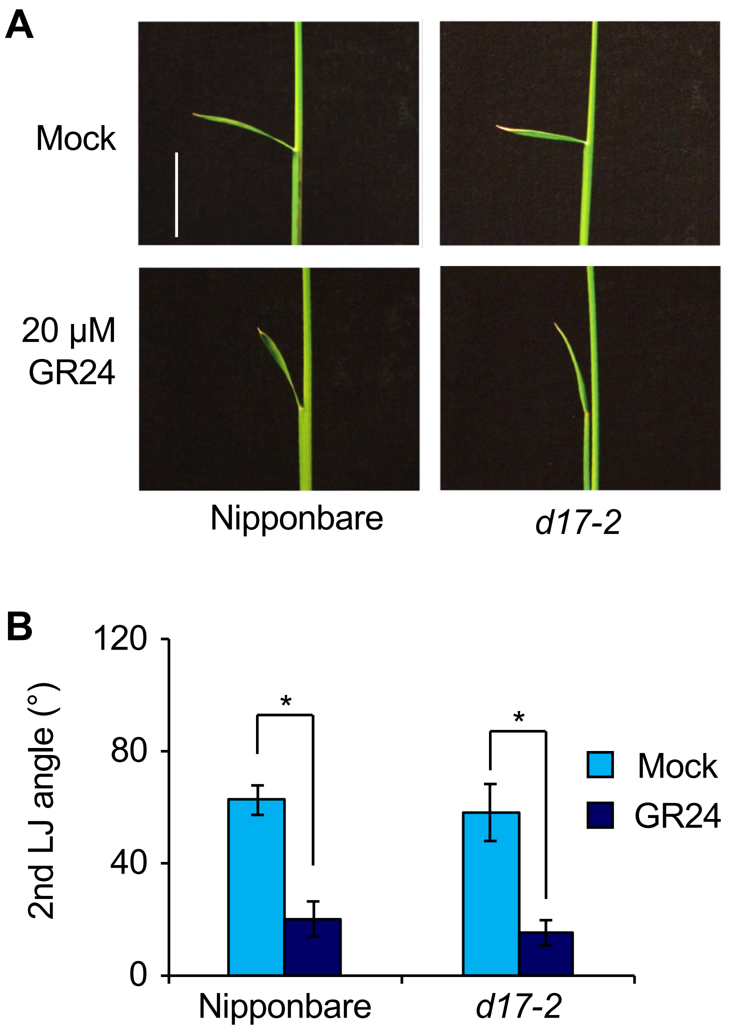

FIGURE S5 | Effect of exogenously applied GR24 on LJ angle in 'Nipponbare'-background rice seedlings. **(A)** Images of the 2nd leaves of 12-day-old seedlings treated or not with 20 μM GR24. Bar, 1 cm. **(B)** Second-LJ angle of seedlings treated or not with GR24. *$P < 0.05$ (Student's *t*-test). Data are means ± S.E. ($n = 5$; eight seedlings per experiment).
